# Nanoparticles of banana peels as a potential source of bioactive compounds and their activities on HepG2

**DOI:** 10.1038/s41598-025-93382-x

**Published:** 2025-04-05

**Authors:** Donia S. Hassanin, Sahar R. Abdelhady, Adel Kh. Ghazi, Waleed Z. Badawy

**Affiliations:** Department of Food Technology, Faculty of Agriculture, Kafrelsheikh University, Kafr El-Shaikh, Egypt

**Keywords:** Banana peels (*Musa acuminata*), Superfine grinding (ball milling), Bioactive compounds, Antioxidant, Antimicrobial, Anticancer, Biotechnology, Cancer, Cell biology, Microbiology, Structural biology, Environmental sciences, Diseases, Nanoscience and technology

## Abstract

Nanoparticles of blanched green banana peels (BGBP) were prepared using physical technique (by grinding) in order to avoid any harm effect on human health that could occurred when metals were used for preparing nanoparticles size (NPs) of banana peels. This work was designed to study the preparation of nano scale (70–135 nm for TEM) (243.4–933.9 nm for SEM) and normal size (0.12–0.25 µm for TEM) (1.150 µm for SEM) from BGBP after milling and evaluate the activities of their extracts as antioxidant, antimicrobial and anticancer agents. The size and shape of nanoparticles were analyzed using Scanning Electron Microscope (SEM) and it cleared the appearance of particle aggregation was attributed to mechanical pressure and friction resulting from the interaction between the abrasive balls and the pulverizing vessel’s inner surface. Also, Transmission Electron Microscope (TEM) shows presence of different spherical shapes ranging between 70 and 135 nm, along with the emergence of slender fibrillary shapes., on the other hand, Fourier transform IR (FT-IR) cleared that the higher extraction yields of phenolic compounds and greater antioxidant activities were achieved due to the increased surface area of nano-scale samples following milling. In addition, X-ray diffraction (XRD) determined the materials crystalline structure. Bioactive compounds (mainly phenolic compounds) were recovered by extracting banana peels weather from normal size or (NPs). The extracted bioactive compounds were subjected to evolution as antioxidant, antimicrobial and anticancer agents. Dealing with this study, it was concluded that bioactive compounds extracted from NPS of BGBP showed antioxidant, antimicrobial and anticancer activities higher than those extracted from the normal size ones. So, it is strongly recommended to use NPs of BGBP for producing these bioactive compounds since these compounds are important to protect humans against a lot of dangerous diseases. Finally, the potential applications of these compounds in the pharmaceutical or food industries would be beneficial.

## Introduction

Banana is the largest herbaceous plant globally, ranks among the top ten vital crops, occupying the fourth spot in global production with over 7 million tons harvested through traditional farming methods. Roughly^1^. In addition, approximately 90 million tons of bananas are grown in tropical regions like Africa (13%), South and Central America (28%), and South Eastern Asia (47%). However, 40% of this yield is discarded, mostly in the form of banana peels^2^. On the other hand, the average consumption of bananas per person is 12 kg per month, making it the leading food crop in the world after rice, wheat and corn. Consequently, here has been a significant rise in worldwide banana production over the last two decades, reaching approximately 117 million tons in 2019 from around 70 million tons in 1999^3^.

Innovative approaches are needed to develop safe and powerful new and eco-friendly technologies, aiming for the advancement of green technology that plays a major role in environmental sustainability by producing nanostructures safely for both human health and the environment^4–6^. Nanotechnology refers to the designing, synthesis, and use of particles ranging from 1 to 100 nm in size, leading to a variety of nano-scale materials that improve solubility and bioavailability, reduce toxicity, enhance tissue macrophage circulation, and protect against physical and chemical degradation^5^. Nanoparticles possess a notable benefit because of their elevated ratio of surface area to volume, which enhances their surface reactivity and changes the physical and biological properties of nanomaterial’s^6,7^.

There are many physical methods that are widely used to produce nanomaterials, among these methods are inert gas condensation (IGC), physical evaporation, electric arc discharge, sputtering, and laser methods. Many characterization analysis techniques of nanomaterials, including ultraviolet–visible (UV–V) spectroscopy, XRD (X-ray diffraction), BET (Brunauere emmette teller), FESEM (Field emission scanning electron microscopy), FTIRS (Fourier transform infrared spectroscopy), TEM (Transmission electron microscopy) and Zeta size analysis. The unique properties that distinguish nanomaterials, allows them to penetrate many applications that directly serve the world. Nanomaterials have been utilized in various applications in the environment, agriculture, food industries, medical industries, chemical processing, and military industries^8^.

Banana peels are rich in their content of dietary fiber (50% on a dry matter basis), proteins (7% dry weight), and essential amino acids^9^. Furthermore, the significant amount of organic material found in banana peels, including fats, proteins, and carbohydrates, indicates that they are a valuable source of fiber and carbohydrates. Nevertheless, the considerable amount of fiber found in banana skins can aid in treating constipation and enhancing one’s overall health and well-being^10^. Also, Banana peels contain high levels of phytochemical compounds, particularly antioxidants. The concentration of phenolic compounds in banana peel varies between 0.90 and 3.0 g per 100 g of dry weight^11^. The peel was particularly high in phenolic, with a concentration of 907 mg/100 g dry weight. It additionally has antioxidant substances like polyphenols, catecholamines, and carotenoids^12^. These phytochemicals have been shown to exert important biological effects (antibacterial, antihypertensive, antdiabetic, anti-inflammatory activities and anticancer). Thus, the presence of these important and biologically active substances in banana peels indicates that the peels possess valuable medicinal potential that should be explored and utilized^13^.

In addition, bananas contain various antimicrobial compounds such as dopamine, gentisic acid, ferulic acid, lupeol, and 3-carene, which give them natural antioxidant and antimicrobial properties. It provides a simple and environmentally friendly alternative to chemical antibacterial substances and fungicides^14^. Interestingly, some fruits, such as banana peels, have shown higher antimicrobial activity in their seeds and peels compared to the pulp^15^. The use of peels as antimicrobial agents can help restore current environmental waste problems and at the same time bring enormous benefits to humanity^16^. Also, various compounds produced by banana peel such as flavonoids and polyphenols have shown anti-cancer activity. Flavonoids are a class of compounds known to have antioxidant and anti-cancer activity. Polyphenols can prevent carcinogenesis by affecting molecular pathways in the initiation, promotion, progression, and metastasis stages^17^.

This study was initially conducted to investigate if it is feasible to produce banana peel nanoparticles through a physical method (grinding) as opposed to a chemical method, which may have negative effects on human health with prolonged usage. The second aim of this research is to determine how altering the particle size of banana peels powder from normal to NPs impacts the antioxidant, antimicrobial, and anticancer properties of their bioactive extracts. The size and shape of nanoparticles were analyzed using Scanning Electron Microscope (SEM), Transmission Electron Microscope (TEM), Fourier transform IR (FT-IR) and X-ray diffraction (XRD).

## Materials and methods

### Materials

#### Plant material

Bananas (*Musa acuminata*) were purchased in their second stage of maturity, when they were light green without the use of ethylene, from the Faculty of Agriculture farm at Kafr El-sheikh University, located in Kafr El-sheikh Governorate, Egypt.

#### Chemicals and solvents

All chemicals were pure (1, 1-diphenyl-2-picrylhydrazyl (DPPH), acetic acid (0.5%), acetonitrile (99.5%) and ascorbic acid and solvents used in this study purchased from El-Gomhoria Company for Chemicals and Drugs at Tanta City, Egypt.

#### Microorganisms

All strains of the microorganisms were kindly provided by the Department of Plant Pathology at Kafrelsheikh University Faculty of Agriculture. We examined these microbes to ensure their authenticity and purity. In addition to gram negative *Escherichia coli* (ATCC 8739) and *K. pneumonia* (ATCC13883), a bacterial strains representing gram positive *Bacillus Subtilis* (ATCC 6633) and *Staph.aureus* (ATCC 6538). *Aspergillus niger* was used to represent molds in this experiment, *C. albicans* (ATCC 10221) as a representation of yeasts, the original cultures were stored in 10% glucose and new cultures were regularly created every two months. The cultures were stored in a refrigerator at 5°C until used.

### Methods

#### Preparation of sample

Bananas “Grande Nain” cultivar *(Musa acuminata)*, were obtained from the farm of the Faculty of Agriculture, Kafr El-sheikh University, Egypt. Fruits were selected according to uniformity and level of maturity (stage 2—light green). The fruits were weighed, washed with distilled water, then peeled and the peels were cut into homogeneous parts. They were immersing in water at 100 °C for 6 min. Then immediately immersed in a 0.5 percent solution of sodium metbisulfite for 15 min to prevent browning. The peels were placed in separate stainless steel trays and air-dried at 50 °C in an air oven until a constant weight was obtained (about 17 h). The resulting dried material is crushed, ground, and sift using 100 mesh screen sieve, then stored in polyethylene plastic bags at a temperature of 25 °C until used.

#### Preparation of BGBP and NPs extracts

The dried of BGBP and NPs samples (1 g) were individually placed in 10 ml of distilled water, 70% methanol, 70% ethanol, and 70% acetone for 24 h at room temperature. The extraction process involved filtering every mixture with Whatman No. 1 filter paper three times. The filtrate was evaporated to dryness at 40 °C using a rotary evaporator. The unrefined extracts were kept in a fridge until they were analyzed.

#### Gross chemical composition

Moisture, ash, ether extract, crude fiber and crude protein were performed according to the methods given^18^. The total carbohydrates amount was determined by subtracting protein, ether extract and ash from the 100% total mass, as reported^19^. The aforementioned tests were all conducted three times, and the findings were reported as an average based on the weight of the samples after they were dried.

#### Total phenolic content

The total phenol (TP) content was measured using the Folin Ciocalteu reagent assay and gallic acid was used as the standard^20^. 9 ml of distilled water was placed in a 25 ml volumetric flask along with 1 ml each of BGBP and NPs extracts. After the addition of one milliliter of Folin Ciocalteu’s phenol reagent, the mixture was shaken. After five minutes. The blend was mixed with 10 ml of Na_2_CO_3_ solution containing 7% concentration (w/v). The solution was thinned out and then combined with distilled water to reach a total volume of 25 ml. After 90 min of incubation. The spectrophotometer (Unicum UV 300) was used to measure absorbance at 750 nm with the blank set as the detector at room temperature. The amount of phenolic content in samples was measured in milligrams of gallic acid equivalents (GAE/g dry weight). Samples were analyzed in triplicates.

#### Total flavonoid content

Total flavonoid (TF) was determined by the aluminum chloride method using quercetin as a standard^21^. 4 ml of distilled water was first added to a 10 ml volumetric flask, and then 1 ml of BGBP and NPs of BGBP extracts were added. 0.3 ml of 5% NaNO_2_ was added to the flask, followed by the addition of 0.3 ml of 10% AlCl_3_ after 5 min. 6 min later, 2 ml of 1 M NaOH was introduced and the volume was topped up to 10 ml with distilled water. The mixtures were fully mixed and the level of absorption was measured at 510 nm using a spectrophotometer (Unicum UV 300) compared to a reagent blank that had been prepared. The concentration of flavonoids in the sample was indicated in milligrams of quercetin equivalents (QE) per gram of dry weight. Samples were analyzed in triplicates.

#### Total tannins content

Total tannin (TT) was measured using the Folin-Ciocalteu reagent^22^. 7.5 ml of distilled water was mixed with 1 ml of BGBP and NPs of BGBP extracts, then 0.5 ml of Folin reagent and 1 ml of 35% sodium carbonate solution were added. The diluted solution was mixed with distilled water to reach a volume of 10 ml, and then the absorbance was taken at 775 nm using a Unicom UV 300 spectrophotometer while comparing it to a blank reagent. The amount of tannins in the sample was determined in milligrams of tannic acid equivalent (TE) per gram of dry weight. Samples were analyzed in triplicates.

#### Identification of phenolic compounds by HPLC

Phenolic compounds in BGBP and NPs of BGBP acetone extracts were identified using HPLC^23^. The HPLC system includes an Agilent 1100 series with a DAD detector (G1315B) and DEGASSER (G1322A). 5 μl sample injections were carried out using an Agilent 1100 series auto-sampler. The column employed for the chromatographic separations was the ZORBAX-EclipseXDB-C18 (4.6 × 250 mm, particle size 5 μm). A steady 1 ml/min flow rate was employed with the following mobile phases: (A) distilled water with 0.5% acetic acid and pH 2.65; and solvent (B) 99.5% acetonitrile with 0.5% acetic acid. The DAD detector was set at wavelength 280 nm for 50 min, eluting linearly from A to B. The identification of phenolic compounds in acetone extracts of BGBP and NPs of BGBP was done by comparing their retention times with a standard mixture chromatogram. Peak area measurements were used to determine the concentration of a single compound.

#### Converting BGBP particles from normal size (70–135 nm) and nano-scale (1–100 nm) using ball milling

Ball milling (BM) is a green technology for the preparation and functionalization of nano derivatives^24^. BM was carried out employing the Planetary Micro Mill (Pulverisette 7 classic line, FritschGmbH, Germany). BGBP was placed in a stainless-steel container with Zirconium dioxide grinding balls, which were 5–10 mm in diameter. The BM process was carried out in an argon gas environment to avoid the impact of milling heat, with a powder mass to ball mass ratio of 1:7 by weight. The high-speed milling operated at a speed of 500 revolutions per minute (rpm) for 5 h^25^.

#### Scanning electron microscopy (SEM)

The physical structure and shape of banana peel powder were characterized using a JSM-7500F high-vacuum Scanning Electron Microscope (SEM) (Jeol Ltd, Japan). The specimen was placed on the metallic mount and gilded, enabling the samples to be prepared and moved into the microscope chamber in a vacuum. Following this, SEM was utilized to take images of the powder with a 15 kV acceleration voltage^26^.

#### Transmission electron microscope (TEM)

In TEM, a small amount of BGBP and NPs of BGBP in water was placed on a copper grid with carbon coating, then left to dry at room temperature. The images were captured using a TEM (JEOL-JEM-1200 EX, Japan) at 80 kV. The pattern of electron diffraction was also documented for a specific area. The PSS- NICOMP 380-ZLS particle sizing spectrophotometer, located in St. Barbara, USA, was used to determine the mean particle size and particle size distribution^27^.

#### Fourier transform IR analysis (FT-IR)

FT-IR spectral analysis was conducted with a Bruker FT-IR spectrometer (Invenio S, Germany). The IR spectra were collected using a wavenumber range of 400–4000 cm^−1^, with a spectral resolution of 4 cm^−1^ and 64 scans^28^.

#### X-ray diffraction (XRD) measurement

The XRD diffractometer used to analyze XRD patterns of fibers, cellulose, and CNCs was the PANalytical Xpert pro MRD diffractometer in Amsterdam, Netherlands, operated at 40 kV and 30 mA with Cu Ka radiation at 0.15406 Å wavelength and a nickel monochromator filtering wave. The specimens were scanned at room temperature with a scanning rate of 0.4°/min across the range of 20 = 5–40°. The equation used to determine the crystallinity index (CI%) was calculated as follows^29^.

$${\text{CI}} = \frac{{{\text{I2}}00 - Iam}}{I200}$$

where I200 represents the intensity at level 200, while Iam is the lowest intensity between levels 110 and 200, specifically at the peak of 20 to 21 and 9. 1200 is made up of both the crystalline and amorphous sections, whereas Iam specifically denotes the amorphous portion. The Scherer equation was utilized to determine the crystallite size of BGBP and NPs^30^.

$${\text{D}} = \frac{K\lambda }{{\beta_{{{1}/{2}}} cos\theta }}$$

where K is the Scherrer constant (0.94), λ is the X-ray wavelength (λ = 0.15406 Å), β_1/2_ is the full width at the half maximum (FWHM) of the XRD peak, and θ is the Bragg’s angle.

The size of the crystallite was determined by analyzing the diffractograms and removing the impact of the amorphous peak (Iam) from the peak height of 200 planes.

#### Determination of antioxidant activity by DPPH of BGBP in normal size (70–135 nm) and nano-scale (1–100 nm)

The antioxidant capacity of various banana peel extracts was assessed using 1, 1-diphenyl-2-picryl hydrazyl (DPPH) as the indicator. To summarize, a solution of 0.1 mM DPPH in ethanol was made. 1 ml of this solution was mixed with 3 ml of various ethanol extracts at varying concentrations (3.9, 7.8, 15.62, 31.25, 62.5, 125, 250, 500, 1000 µg/ml). Only the ethanol-soluble extracts were utilized, and different concentrations were made through dilution. The blend was vigorously shaken and left at room temperature for 30 min before measuring absorbance at 517 nm. Through the utilization of a spectrophotometer (specifically the UV–VIS Milton Roy model). The experiment was performed three times using ascorbic acid as the reference standard compound. The concentration of the sample needed to inhibit 50% of the DPPH free radical was determined by calculating the IC 50 value with a Log dose inhibition curve. Higher free radical activity was suggested by lower absorbance in the reaction mixture^31^. The percent DPPH scavenging effect was calculated by using following Equation:

$${\text{DPPH}}\;{\text{scavenging}}\;{\text{effect}}\left( \% \right)\;{\text{or}}\;{\text{Percent}}\;{\text{inhibition}} = {\text{A}}0 - {\text{A1}}/{\text{A}}0 \times {1}00.$$

where A0 was the Absorbance of control reaction and Al was the Absorbance.

in presence of test or standard sample.

#### Determination of antimicrobial activity of BGBP in normal size (70–135 nm) and nano-scale (1–100 nm)

The antimicrobial activity of different samples of normal and NPs of BGBP powder were measured using Disk or well Diffusion Approaches^32^. The agar well diffusion method is commonly employed for assessing the antimicrobial properties of plant or microbial extracts. Just like in the disk-diffusion method, the agar plate surface is inoculated by spreading the microbial inoculum across the entire agar surface. Next, a sterile cork borer or tip is used to punch a hole with a diameter of 6–8 mm aseptically, following which (10mg/ml) of the antimicrobial agent or sample extract or extract solution added to the well. Next, agar plates are placed in an incubator with conditions specific to the type of microorganism being tested. The antimicrobial substance spreads through the agar medium and hinders the growth of the tested microbial strain.

#### Determination of Anticancer activity of BGBP of in normal size and NPS

The cytotoxicity of all the extracts was determined by a tetrazolium (MTT) assay^33^. HepG2 cells were seeded onto 96 well plates at a density of 2 × 105 cells/ml per well in 100 μl of RPMI 1640 using a Bioklenz laminar air flow hood, and incubated for 24 h at 37°C and 5% CO_2_ in a CO_2_ incubator (New Brunswick Galaxy 170S). Following that, the medium was exchanged with a fresh one that had varying concentrations of the sample (31.25, 62.5, 125, 250, 500, and 1000 μg/ml) for an additional 48-h incubation period. Next, each well received 20 μl of MTT stock solution (5 mg/ml in phosphate-buffered solution) and was incubated for 5 h. The solution was eliminated, and 200 μl of DMSO was introduced into each well to dissolve the MTT metabolic product. The ELISA reader measured the absorbance at 560 nm. The formula was used to determine the percentage viability of the samples.

$$\% \;{\text{cytoviability}} = {\text{A}}_{{{56}0}} \;{\text{of}}\;{\text{treated}}\;{\text{cells}}/{\text{A}}_{{{56}0}} \;{\text{of}}\;{\text{control}}\;{\text{cells}} \times {1}00\% .$$

#### Statistical analysis

The received data were statistically analyzed^34^. All tests were repeated triplicate. The data was assessed for statistical significance using one-way analysis of variance (ANOVA) followed by Duncan’s multiple-range test, with a significance level of *p* < 0.05. Statistical analysis was conducted with SPSS version 23.0 for all data.

## Results and discussion

### Proximate chemical composition of BGBP

Table 1 showcased the nutritional content of BGBP agro-wastes in this research.

**Table 1:** Proximate chemical composition of BGBP powder (Percentage were calculated on dry weight basis).

Components	Concentration (%)
Moisture**	91.21 ± 0.04
Ash	13.42 ± 0.73
Ether extract	3.98 ± 0.11
Crude fibers	28.74 ± 0.29
Crude Protein	9.06 ± 0.06
*Total carbohydrates	73.54 ± 0.9

All values in Table are means of triplicate determinations ± standard deviation (SD).

* Total carbohydrates were calculated by difference. **: On wet weight basis.

Proximate analysis provides important data on the nutritional content and aids in evaluating the sample’s quality. It gives details about the levels of moisture, protein, ether extract, ash, fiber, and carbohydrate. Research has indicated that fruit wastes, including peels and seeds, contain a significant amount of essential nutrients such as carbohydrates, proteins, ether extract, fibers, and phytochemicals^35^. It should be noted from Table 1 that moisture content of the sample was relatively high (91.21%) and close enough to those found by^36^. In addition, it should be noted also that banana peels were rich in carbohydrates. Furthermore they have moderate amounts of crude fiber, ash and crude protein. Meanwhile, they seem to have a low level of ether extract. Banana peels are rich in their content of dietary fiber (50% on a dry matter basis), proteins (7% dry weight), and essential amino acids^9^. Also, the results obtained align with previous findings^9,37,38^.

### Total phenols (TP), flavonoids (TF) and tannins (TT) of normal size (70–135 nm) and nano-scale (1–100 nm)

Of BGBP extracts:

Phenolic compounds are important secondary metabolites and are found in higher levels in banana peels compared to other fruits^39^. Table 2 displays the outcomes of TP, TF, and TT in aqueous, 70% methanol, 70% acetone, and 70% ethanol extracts. The information in Table 2 demonstrated that the acetone extract from normal-sized and nano-sized banana peels has the highest concentrations of TP (30.02 and 50.3 mg/g dry weight), TF (31.56 and 55.4 mg/g dry weight) and TT (22.95 and 36.05 mg/g dry weight), respectively. While the aqueous extract showed the lowest values for TP (15.97 and 27.37 mg/g dry weight), TF (18.23 and 28.1 mg/g dry weight), and TT (19.34 and 30.39 mg/g dry weight) respectively. The results obtained indicate that banana acetone extract possesses a high content of phenolic, flavonoids and tannins. Banana showed high content in phenolic compounds, especially vanillic and ferulic acids. Banana acetone extracts found to have considerable antioxidant and antimicrobial activities. Banana leaves acetone extract also presented vigorous activity against breast and liver hepatocellular tumor cell lines^40,41^.

**Table 2:** Total phenols (TP), flavonoids (TF) and tannins (TT) of normal size and nano-scale of BGBP extracts.

Sample	Extracts	TP(mg GAE/g DW)	TF(mg QE/g DW)	TT (mg TE/g DW)
Normal size (70–135 nm)	Aqueous	15.97^c^ ± 0.10	18.23^c^ ± 0.34	19.34^b^ ± 0.23
	Methanol 70%	27.28^b^ ± 0.19	29.14^b^ ± 0.25	19.35^b^ ± 0.11
	Acetone 70%	30.02^a^ ± 0.08	31.56^a^ ± 0.3	22.95^a^ ± 0.08
	Ethanol 70%	27.86^b^ ± 0.15	29.39^b^ ± 0.21	21.30 ± 0.06
Nano-scale (1–100 nm)	Aqueous	27.37^c^ ± 0.40	28.1^d^ ± 0.08	30.39^c^ ± 0.15
	Methanol 70%	42.15^b^ ± 0.34	43.69^c^ ± 0.17	31.80 ± 0.76
	Acetone 70%	50.3^a^ ± 0.28	55.4^a^ ± 0.89	36.05^a^ ± 0.11
	Ethanol 70%	44.28^b^ ± 0.33	46.58^b^ ± 0.28	33.18^b^ ± 0.18

All values represented as mean ± S.D Means with different letters are significantly different (*p* < 0.05).

Nevertheless, ethanol yielded good outcomes for extracting BGBP and NPs, with TP content measuring (27.86 and 44.28 mg/g DW), flavonoids content measuring (29.39 and 46.58 mg/g DW), and tannins measuring (21.30 and 33.18 mg/g DW), respectively. The methanol extract in this study showed phenolic content at 27.28 and 42.15 mg/g DW. These results exceed the ones he achieved^42,43^**.**

### Identification and quantification of phenolic compounds of normal size (70–135 nm) and nano-scale (1–100 nm)

**Fig. 1:**
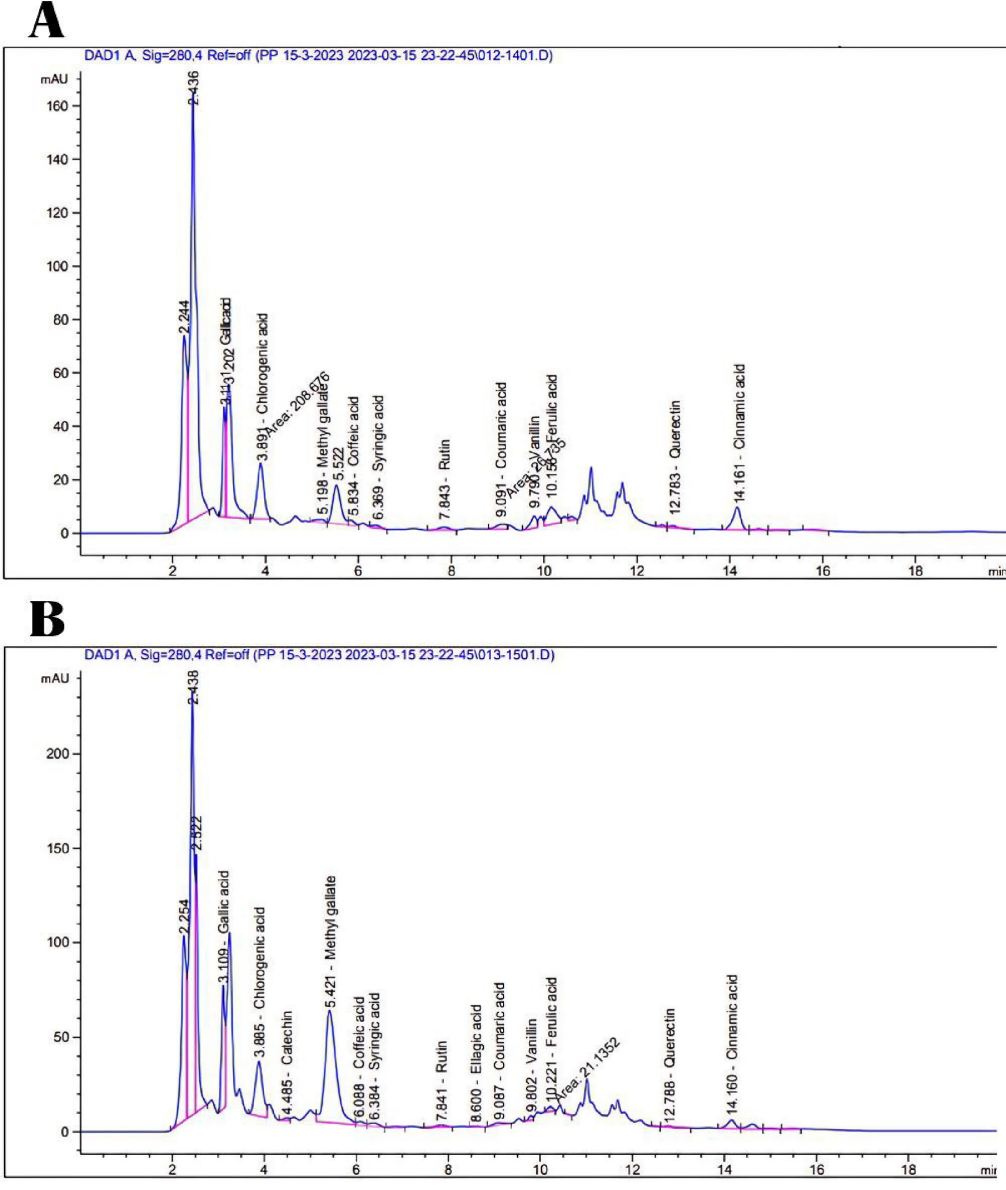
HPLC chromatogram of BGBP in normal size (**A**) and nano-scale (**B**).

Data in Fig. 1 and Table 3 indicate that the levels of specific phenolic compounds were extracted using a 70% acetone extract. Variations in diversity, environmental factors, and farming methods have a significant impact on the composition and levels of phenolic compounds. Yet, the drying procedure could impact the composition of the matrix components and their ability to promote biological activity. The separation and identification of phenolic compounds in normal size and NPs of banana peels were analyzed by high-performance liquid chromatography (HPLC). 11 phenolic compounds identified in banana peels both in normal size and NPs extracted using acetone include gallic acid, chlorogenic acid, methyl gallate, coffeic, syringic acid, rutin, coumaric acid, vanillin, ferulic acid, querectin and cinnamic acid. Ellagic acid and catechin are found in NPs banana peels, but are not present in normal size banana peels this is a result of the ball grinding process. The ultra-fine grinding led to an increase in the concentrations of Gallic acid, chlorogenic acid, methyl gallate, querectin, coffeic acid, syringic and rutin. Nevertheless, the levels of coumaric acid, vanillin, ferulic acid, and cinnamic acid decreased as a result of the grinding procedure. The results obtained align with previous findings^44–47^.

**Table 3:** Quantification of the main phenolic compounds present in acetone extracts (ug/ml crude extract) by HPLC.

Compounds	Normal size (70–135 nm)	Nano-scale (1–100 nm)
Gallic acid	28.58	54.21
Chlorogenic acid	15.3	27.39
Catechin	–	2.70
Methyl gallate	0.83	44.38
Coffeic acid	1.38	1.65
Syringic acid	0.92	1.54
Rutin	2.24	2.50
Ellagic acid	-	1.10
Coumaric acid	0.84	0.69
Vanillin	1.73	0.62
Ferulic acid	6.38	1.44
Querectin	1.20	1.09
Cinnamic acid	1.85	0.99

### Characterization of nano banana peel particles

#### SEM characterization

**Fig. 2:**
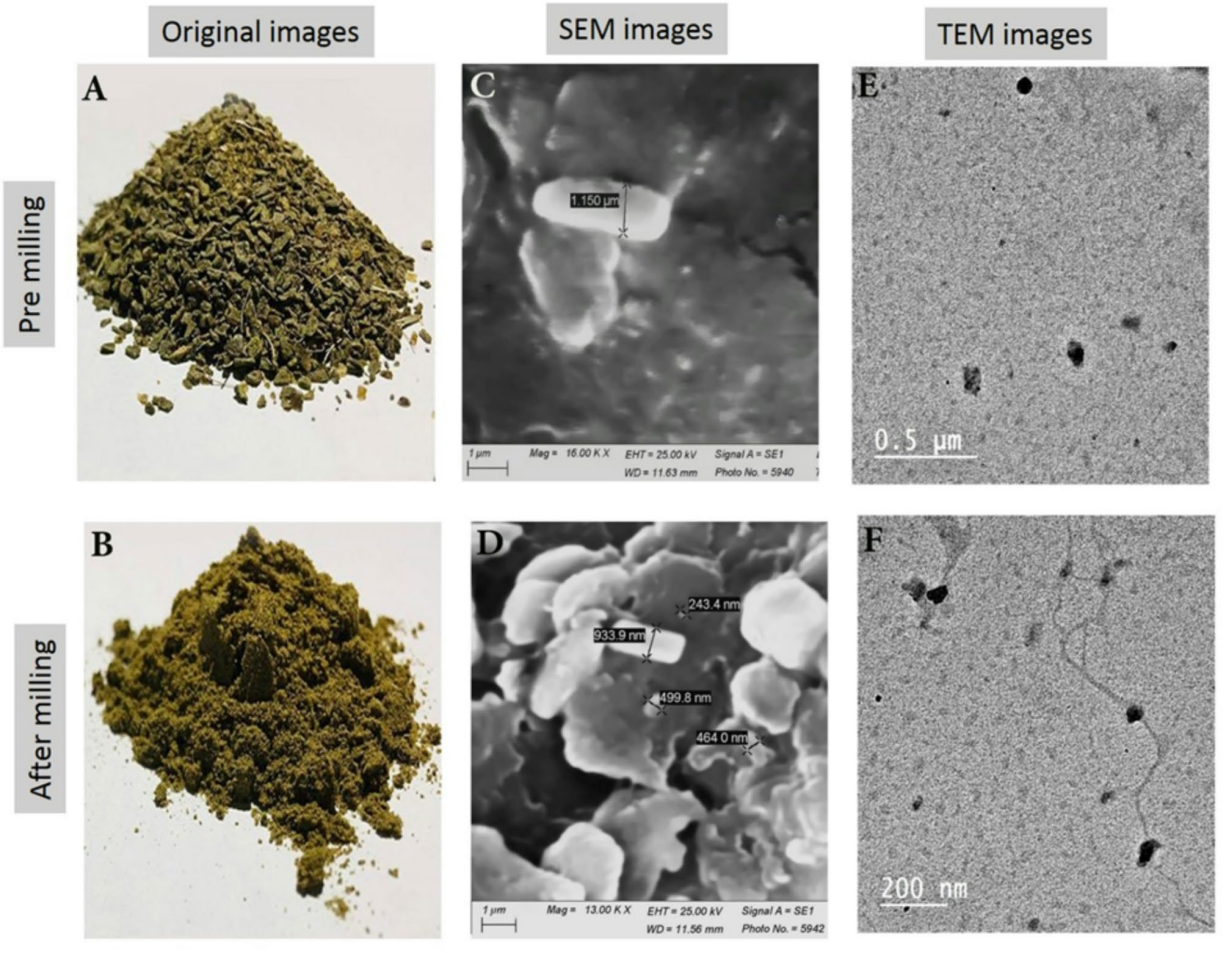
Original images (**A**, **B**), SEM images (**C**, **D**) and TEM images (**E**, **F**) of BGBP pre and after milling.

SEM micrographs of the prepared particles and originals images are shown in Fig. 2C, D. The SEM images of NPs of BGBP powders revealed that the particles underwent fragmentation and developed sharp edges in consequence of the mechanical forces applied during BM. It also showed that the particles had a spherical shape, although some particles showed irregular shapes^48^. The appearance of particle aggregation was attributed to mechanical pressure and friction resulting from the interaction between the abrasive balls and the pulverizing vessel’s inner surface. Compared with pre-milling and after milling originals images as shown in Fig. 2A, B. There are some findings suggest that BM induced ultrafine grinding can modify original surface structures, which, in turn, may affect the physicochemical characteristics of powdery materials^49^. The results^50^ support our findings.

#### TEM’s characterization

The TEM analysis of banana peel powder is depicted in Fig. 2E, F, both before and after grinding with a BM. In Fig. 2E, the typical BGBP size ranges from 0.12 to 0.25 uM. Figure 2F shows clear presence of different spherical shapes ranging between 70 and 135 nm, along with the emergence of slender fibrillary shapes. Compared with pre-milling and after milling originals images as shown in Fig. 2A, B. They vary from extremely small nanofibers to elongated nanofibers. Due to their rounded shape and magnetic characteristics, these nano-sized particles show significant potential for use in areas like medicine, food, and nanotechnology^51^. Take note of the significance the properties and behavior of nanoparticles or nanofibers are significantly affected by their size and shape^52^.

#### Fourier transform IR analysis (FT-IR)

**Fig. 3:**
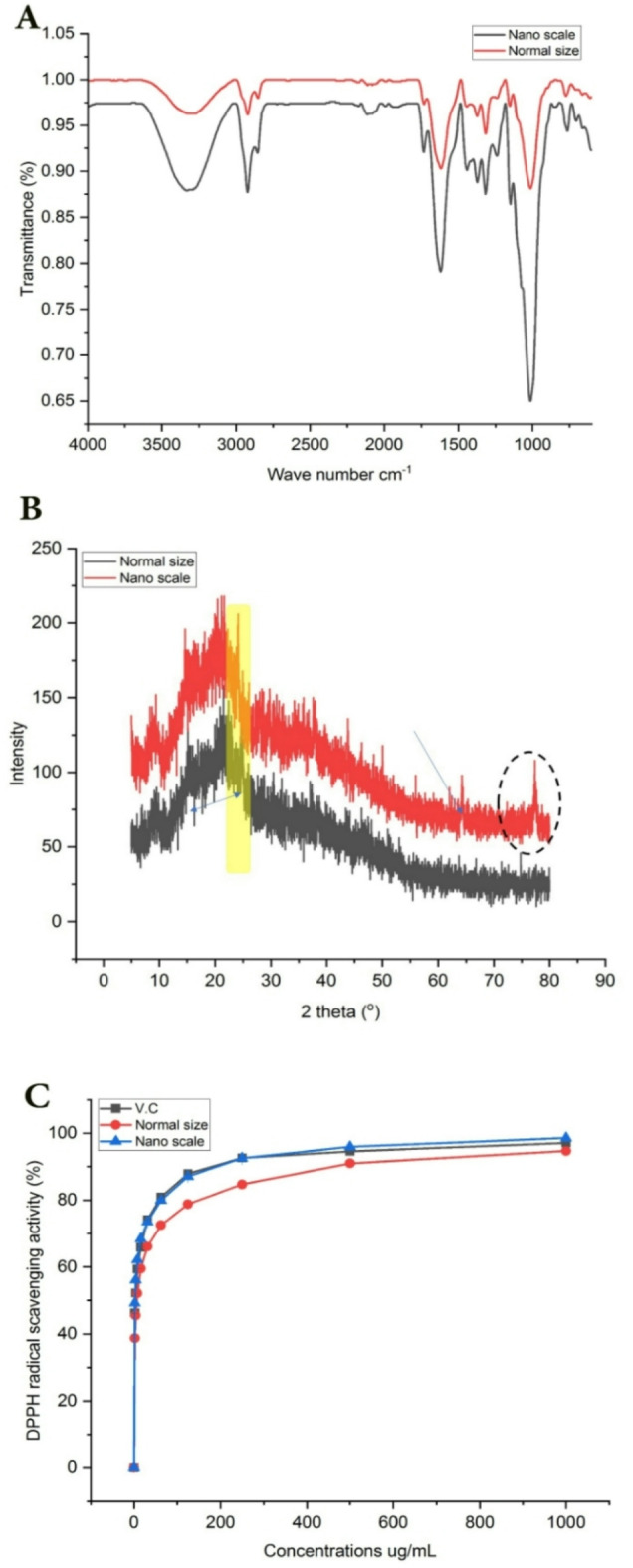
FT-IR (**A**), XRD (**B**) and DPPH (**C**) radical scavenging activity of BGBP in normal size and nano scale.

Figure 3A displays the findings of FT-IR analysis conducted on both normal and nano-sized BGBP extract. Fourier-transform infrared spectroscopy was used to identify alterations in the functional metabolites in both regular and nano-sized BGBP by examining characteristic IR peaks. Both samples exhibited peaks in their spectra at around 3296.35 and 3302.03 for regular size and nano-scale, respectively. Typically, the bands from 3500 to 3200 cm^−1^ are associated with OH stretching vibrations of phenolic structures^53^. Peaks in the range of 2918–2851 cm^−1^ are likely caused by stretching vibrations of CH and CH_2_. The peak observed at 1638 cm^−1^ is indicative of alkene C=C stretching vibrations. The band observed at 1034 cm^−1^ was attributed to the stretching vibrations of C–O in lignin and cellulose^26^. The nano-scale sample at 3302.03 cm^−1^ showed a slight increase in its peak spectrum when using ball-milling technique compared to the other spectrum, possibly caused by the aggregation of phenolic metabolites due to cellulose structure degradation. Moreover, certain peak transmittances like the peaks at 2087.22, 2916.23, and 2856.93 cm^−1^ associated with CH, CH_2_, and OH stretching vibrations were observed and altered following the milling process. This could be the result of milling processes improving the breakdown of hydroxyl groups in cellulose and hemicellulose, leading to the formation of soluble metabolite aggregates and expanding the porosity and external surface area^54^. The higher extraction yields of phenolic compounds and greater antioxidant activities were achieved due to the increased surface area of nano-scale samples following milling. The peak observed at 1012 cm^−1^ was identified as originating from the C–O stretching vibrations of lignin and cellulose. When comparing the spectra of normal and nano-scale, the absorption peaks at 1188, 1248, 1322 and 1625 cm^−1^ were altered due to smaller particle size. The results obtained align with previous findings^26^.

#### Measurement of X-ray diffraction (XRD)

The crystalline structure of BGBP was examined through XRD testing before and after grinding, and the results can be seen in Fig. 3B. Clear differences were observed in the crystalline structure of the powder pre and post grinding. A broad diffraction pattern was found at 23.03° for the NPs, distinct peaks were evident in the powder’s BM 2θ at around 64.56° and 78.04°, showing a crystalline section that matches cellulose’s usual crystalline pattern^55^.

### Antioxidant activity of normal size (70–135 nm) and nano-scale (1–100 nm) of blanched green banana peels extract

Results of antioxidant activity of normal and NPS of BGBP extract are shown in Fig. 3C. It should be noted that antioxidant activity of normal and nano BGBP samples extract increased with increasing the concentration of samples extract, Concentrations ranging from 0 to 1000 μg/mL have been utilized. This in turn helped to increase the yield of the bioactive compounds extracted from the peels. These results were supported by those of^56^, who reported that banana peels are rich in antioxidant compounds such as vitamin C, vitamin E, β -carotene and anthocyanin.

Apparent also from the same figure that nanoparticles of banana peels extract had antioxidant activity higher than that of normal particles extract and V.C, The IC_50_ percentage was 2.09, 5.99 and 2.45 ug/ml respectively^57^, who reported that Nanoparticles exhibit superior DPPH radical scavenging abilities compared to the extract from larger particles, indicating that decreasing the size of the extract enhances its antioxidant properties.

### Antimicrobial activity of normal size (70–135 nm) and nano-scale (1–100 nm) of BGBP extract

**Fig. 4:**
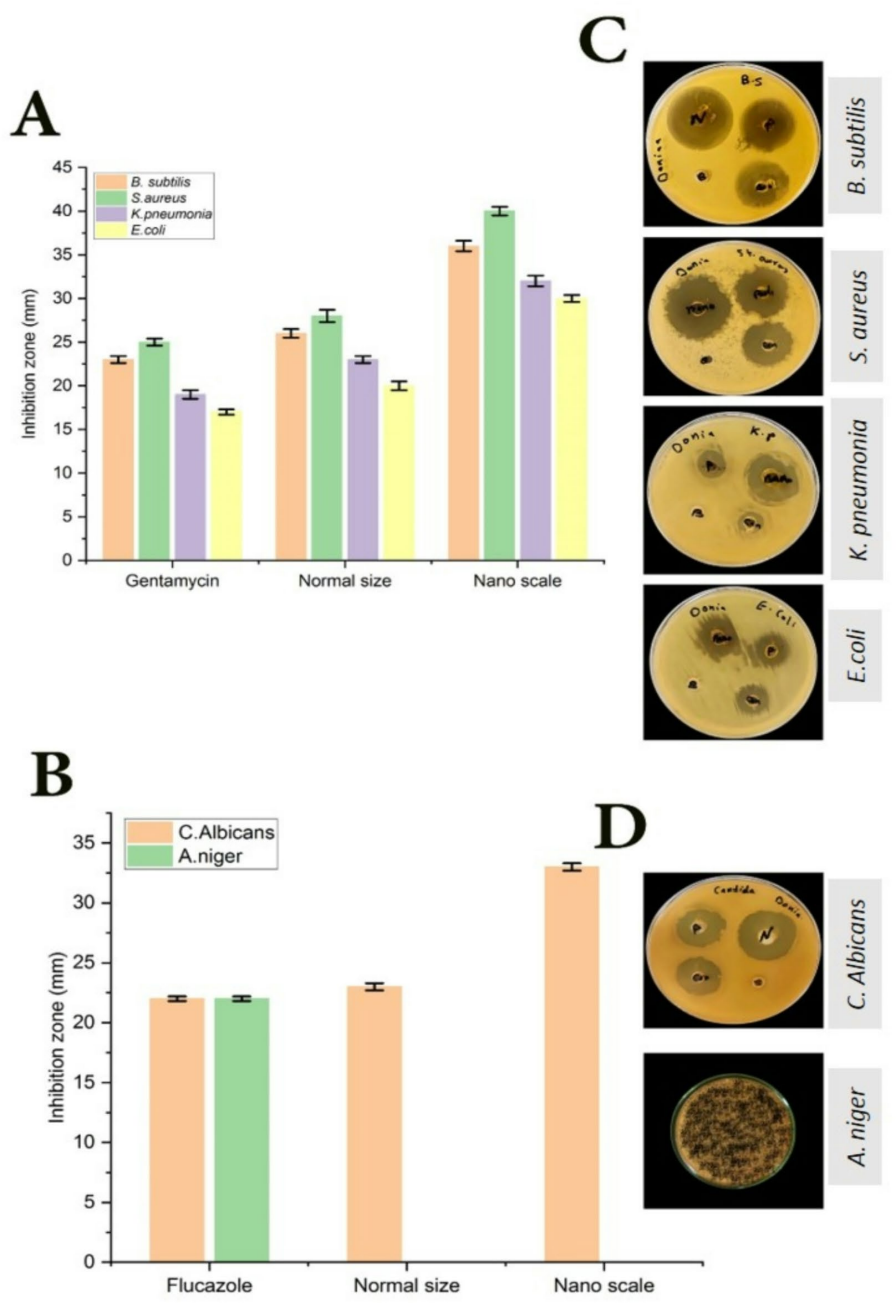
Antimicrobial activity (**A**, **B**), Zone of inhibition of bioactive compounds extracted from normal and NPS of BGBP against various pathogenic bacteria, *C. albicans* and *A. niger* (**C**, **D**). Where: P: Normal size of BGBP. B: Blank (solvent). N: Nano particles size of BGBP. Con: In the case of bacteria, the antibiotic is gentamycin, and in the case of fungi, the antibiotic is flucazole.

The antibacterial activity of starting materials (PPE of BGBP and NPs of BGBP) toward Gram-positive and Gram-negative bacteria were assessed Fig. 4A–C. Compared with the antibiotic Gentamycin was used as a bacterial standard. The results revealed that BGBP nanoparticles at a concentration of 10 mg/mL showed promising antibacterial properties against *B. Subtitles, S. aureus, E. coli* and *K. pneumoniae* where the inhibition zones were 36, 40, 30 and 32 mm respectively. Moreover the lethal effect on gram positive (*B. Subtitles and S. aureus*) was higher than that of gram negative (*E. coli and K. pneumoniae*). *S. aureus* showed the highest sensitivity to NPs of BGBP, whereas *E. coli* displayed the lowest sensitivity. These results were in agreement with those of^58^, and were inconsistent with^59^.

The mechanism of action of NPs of BGBP can be attributed to electrostatic interaction that damages cell membranes, deactivates proteins and enzymes, generates ROS and oxidative stress, binds to proteins causing disruption in homeostasis (electron transport chain), affects signal transduction and Restraint, DNA damage^60,61^. Furthermore, the effectiveness of damaging the cell wall by the nanoparticles is attributed to the roughness of their outer surface. This leads to nanoparticles penetrating the plasma membrane and inducing toxicity in bacteria., as observed by^62^. The result^63,64^ supports our findings.

Apparent also from the same figure that the extracted bioactive compounds that yielded from the nano banana peels had effect on gram positive and negative bacteria higher than those extracted from the normal ones this is shown in Fig. 4A–C. This could be attributed to the high yield of bioactive compounds extracted from the NPS compared to those extracted from the normal ones, which in turn could be related to the high surface area of the NPS. These results were in agreement^27^.

The antifungal activity of BGBP and NPs towards *C. albicans* and *A. niger* was shown in Fig. 4B–D. Compared with the Flucazole as fungi standard. The results showed that BGBP and NPs showed antifungal activity against *C. albicans*, as the inhibition zones were 23 and 32 mm respectively. These results were in agreement^64^, while no effectiveness was shown on *A. niger*. The results obtained align with previous findings^59^.

### Anticancer effect of normal size (70–135 nm) and nano-scale (1–100 nm) of BGBP extract

**Fig. 5:**
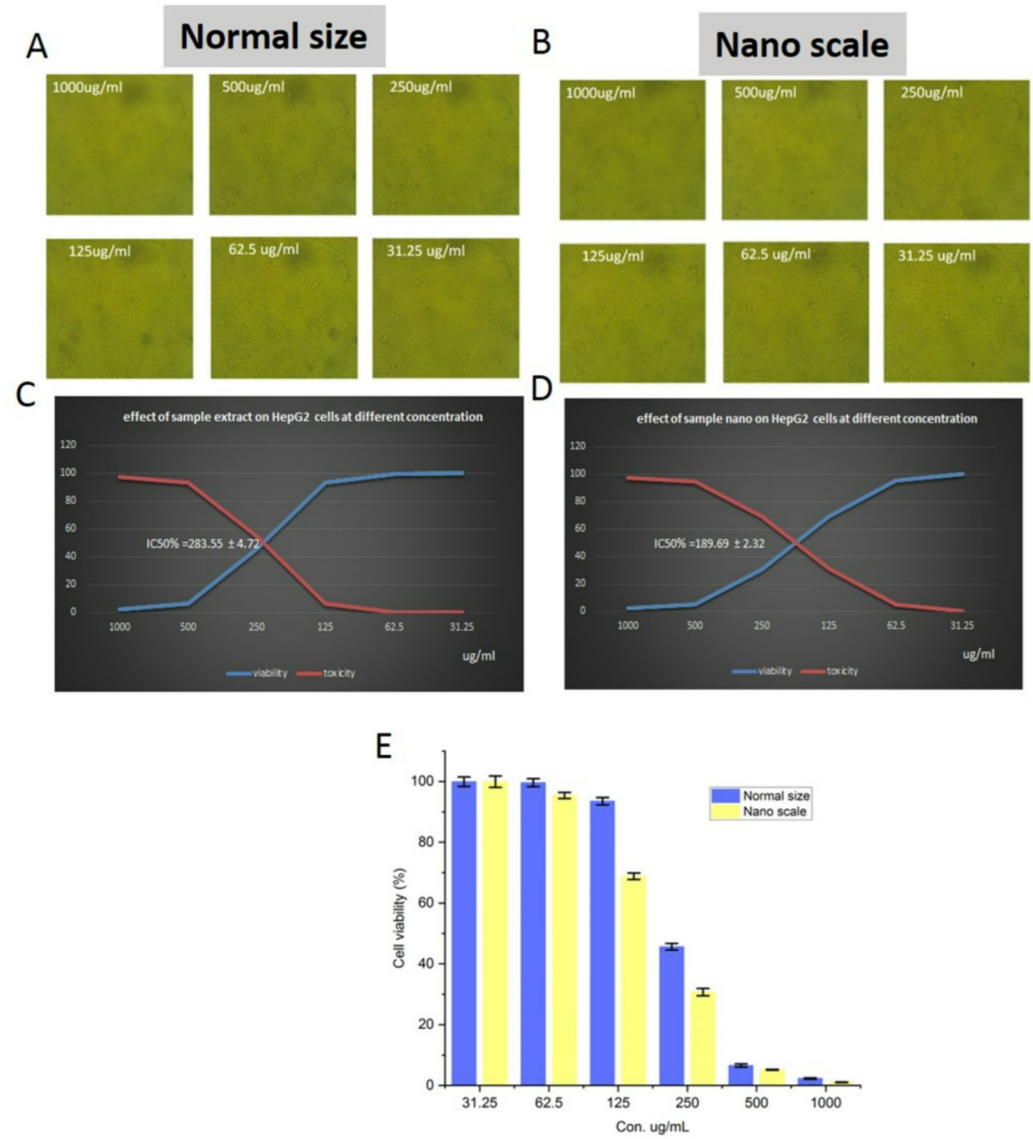
Anticancer activity of BGBP in normal size (**A**, **C**, **E**) and nano-scale (**B**, **D**, **E**). (**A**, **B**) inhibition of cells, (**C**, **D**) IC_50_ (%), (**E**) cell viability (%).

Data shown in Fig. 5 indicated that the blanched normal and NPS banana peels extract illustrated high inhibition effect on the hepatocellular carcinoma cells (HepG2). Cytotoxicity of different concentrations of normal and nano banana peels (31.25–1000 µg/ml) were evaluated. Results indicated that the IC_50_ of bioactive compounds extracted from normal peels was 283.55 µg/ml and nano peels was 189.69 µg/ml. Moreover, the inhibition effect increased with increasing the concentration of banana peels extract, whither from normal or nano peels. In addition the effect of nano banana peels extract was higher than that of normal one. This could be attributed to the high yield of bioactive compounds extracted from blanched green nano banana peels were higher than those of normal one, hence the inhibition effect of the nanoparticles extract was higher than that of the normal one. The results showed that 70% ethanol extract of fruit peel (PBEE) and heart (HBEE) of Raja Bulu banana had median inhibition concentration (IC50) for DPPH scavenging activity at 115.32 µg/mL and 162.52 µg/mL respectively. Overall, the diverse essential micronutrients and phytochemicals found in fruits are largely responsible for their biological activities. These bioactive compounds can potentially stop cancer by functioning as antioxidants, slowing cell growth, triggering cell death, preventing cell invasion, and impacting signaling pathways within cells^65,66^. Nanoparticles with enhanced surface characteristics can diffuse more easily inside tumor cells, thus delivering an optimal concentration of drugs at tumor site while reducing the toxicity. Cancer cells can be targeted with greater affinity by utilizing NMs with tumor specific constituents. Furthermore, it bypasses the bottlenecks of indiscriminate biodistribution of the antitumor agent and high administration dosage. Here, we focus on the recent advances on the use of various nanomaterials for cancer treatment, including targeting cancer cell surfaces, tumor microenvironment (TME), organelles, and their mechanism of action. The paradigm shift in cancer management is achieved through the implementation of anticancer drug delivery using nano routes^41^.

There are many different methods for preparing nanomaterial, nanomaterial are used in many applications in various fields such as the environment, agriculture, industry, medicine, military defense systems, electronics, and energy storage. Regarding future trends, it is the use of nanomaterial in energy storage devices, electronics, environmental protection, and renewable energy production. For example, the use of nanomaterial in developing solar cell systems. Nanomaterial will be used to improve the efficiency of electrodes in energy storage batteries, hydrogen production as a renewable energy source, delivering medicines and treating chronic diseases such as cancer. From the above, the future of advanced technology is closely linked to progress in the field of nanomaterial technology engineering.

## Conclusions

Banana peels that subjected to blanched treatment were treated with ultrafine grinding treatment to change the particles size from normal to nanoparticles size (physical grinding). This technique was used instead of the chemical technique so, the peels will be safe for human consumption changing the particles size from normal to nanoparticles size helped to increase the level of the phenolic compounds extracted from the nano peels, hence increase the activity of nano banana peels as antioxidant, antimicrobial and anticancer agents.

## Data Availability

The data used to support the findings of this study are available from the corresponding author upon request by the same email: walid.metwali@agr.kfs.edu.eg.
